# Organismal Design and Biomimetics: A Problem of Scale

**DOI:** 10.3390/biomimetics6040056

**Published:** 2021-09-28

**Authors:** Valentina Perricone, Carlo Santulli, Francesco Rendina, Carla Langella

**Affiliations:** 1 Department of Engineering, University of Campania Luigi Vanvitelli, Via Roma 29, 81031 Aversa, Italy; 2 School of Science and Technology, Università di Camerino, Via Gentile III da Varano 7, 62032 Camerino, Italy; carlo.santulli@unicam.it; 3 Department of Science and Technology, University of Naples “Parthenope”, URL CoNISMa, Centro Direzionale, Is. C4, 80143 Naples, Italy; 4 Department of Architecture and Industrial Design, University of Campania Luigi Vanvitelli, Via San Lorenzo, 81031 Aversa, Italy; carla.langella@unicampania.it

**Keywords:** biomimetics, bio-inspired design, size, scaling laws, scaling effect, organismal structure and function

## Abstract

Organisms and their features represent a complex system of solutions that can efficiently inspire the development of original and cutting-edge design applications: the related discipline is known as biomimetics. From the smallest to the largest, every species has developed and adapted different working principles based on their relative dimensional realm. In nature, size changes determine remarkable effects in organismal structures, functions, and evolutionary innovations. Similarly, size and scaling rules need to be considered in the biomimetic transfer of solutions to different dimensions, from nature to artefacts. The observation of principles that occur at very small scales, such as for nano- and microstructures, can often be seen and transferred to a macroscopic scale. However, this transfer is not always possible; numerous biological structures lose their functionality when applied to different scale dimensions. Hence, the evaluation of the effects and changes in scaling biological working principles to the final design dimension is crucial for the success of any biomimetic transfer process. This review intends to provide biologists and designers with an overview regarding scale-related principles in organismal design and their application to technical projects regarding mechanics, optics, electricity, and acoustics.

## 1. Introduction

In human history, nature has always been a fascinating model, for both aesthetically attractive forms and functional processes responding to complex adaptive needs, stimulating the research of new expressive and technical solutions. This biological solution transfer is promoted by a multitude of analogies between organisms and artefacts in terms of their components, behaviour, and functions [1,2,3,4]. This is consequential to the fact that both often face similar problems, such as: protection and support (skeleton/frame), ventilation and temperature control in closed spaces (den, beehive, termite mound/environmental conditioning systems), drag reduction (streamline shape of marine animals/hull of boats), and reaction to external stimuli and conditions (nastic movement of plants/dynamic facades) [3,5,6]. Similar problems may be solved by similar solutions; thus, organismal design can provide trustful functional strategies, developed over millions of years of evolution, for the development of novel design inspirations. The interdisciplinary approach that combines the understanding of natural structures, systems and processes with their abstraction and translation into technological applications is known as “Biomimetics” [1,4,7,8,9,10,11,12]. The engineer and physicist, O.H. Schmitt, coined this term in 1957. In 2015, the biomimetic approach was officially introduced and certified by the International Organization for Standardization (ISO 18458).

Historically, important figures in the world of architecture, engineering, design, and biology—such as Leonardo Da Vinci, Antonio Gaudi, Frei Otto, Pierluigi Nervi, Joseph Paxton, Santiago Calatrava, Renzo Piano, Peter Pearce, Julian Vincent, Neri Oxman—have consciously chosen to use and support this bioinspired design approach, which led to original creations and technologies. Nevertheless, even Leonardo da Vinci’s works confirmed how interesting, albeit difficult, this transfer can be. By observing and studying birds, he paved the way to the first construction of a flying machine; however, he was misled because he was unaware that flight was strongly dependent on size; air drag is stronger for a flying machine than for birds, and so the problem lies in how drag and weight are scaled with size [5].

Sometimes in biomimetic transfers, what seems to be effective from a conceptual perspective fails to function in practice. This is mainly due to a lack of consideration concerning organismal design constraints and their dimensional realms. Firstly, in the transfer of solutions, organismal design constraints are a primary consideration [6,13]. Indeed, although organisms can provide profitable inspirations for technical applications, their structural and functional solutions are often neither the most advantageous nor the most adapted in any situation and context. The design of organisms is subjected to different factors, e.g., phylogenetic, functional, morphogenetic, and environmental [6,13]. Secondly, the organismal working principles are based on relative dimensional realms. In nature, mass can vary by more than 27 orders of magnitude [14]. The smallest living organism presently known is a marine archaea, *Nanoarchaeum equitants*, with a size of 400 nanometres, discovered in 2002 in a hydrothermal vent off the coast of Iceland [15]. The largest organisms are the giant sequoia (*Sequoiadendron giganteum* [Lindley] Buchholz), which can reach 70–85 m in height and 5–7 m in diameter [16], and the blue whale (*Balaenoptera musculus* Linnaeus, 1758), which is more than 20 m in length [17]. From the smallest to the largest, every species must respect its size laws. These laws were historically discussed by Thompson in 1917 [18] and recently reorganized in a universal overview and application described as *Universal Scaling Laws* [14]. Although the belief of universal scaling laws has not been totally accepted by the scientific community, it is indisputable that form, process and working principles are interrelated with size since they respond to the physical laws underlying biology [3,5,14]. The examples regarding the influence of scale are structural strength and locomotion, surfaces for oxygen diffusion and food intake, temperature exchange, metabolism, ecological distribution, and the abundance of species [3].

Hence, although organismal design provides excellent mechanical, dynamical, optical, electrical, and acoustic strategies, these bioinspired solutions are applied to products and technologies that often work in different dimensional realms. As a result, a change in scale (i.e., scaling effect) must always be taken into consideration.

In biomimetic literature, very little has been investigated and discussed regarding the problem of scaling biological principles to artefacts. Perez et al. [19] identified and discussed the scaling issue in biomimetic design, reporting five scaling principles. Although representing an acceptable attempt in the identification of the problem and its solution, these principles appear to be vague in their application to all specific biomimetic cases. Moreover, they encourage the transformation of the biological process to achieve the final function (i.e., the conversion of a physical process into a chemical one) [19].

In this regard, if the biological phenomenon loses its efficacy through the dimensional scaling to the final application, the transfer is frequently converted and limited to a vague bioinspiration. Particularly, the biological phenomenon is mimicked using a technology based on different physical working principles. A representative example is the mimicking of the climbing ability of the gecko based on electrical Van der Waals forces [20], which are difficult to scale up to large dimensional applications, by translating them into magnetic or mechanical forces using dipoles or suckers [19]. The real aim of biomimetics to directly mimic the natural system is thus compromised. In this framework, the present review aims to provide an overview regarding the scale-related principles in organismal design and their application at different scales (nano-, micro- and macroscales) in four fields of physics: (1) mechanics, (2) optics, (3) electricity and (4) acoustics. The intention is to offer biologists and designers a reference scenario of biological and biomimetic scaling based on a learning from nature approach. Thus, each paragraph begins with the description of the basic dimensional rules in the biological realm and how they affect the organismal design. Consequently, several reference studies are provided, supporting the scaling in the solution transfer from organisms to manmade artefacts and technologies. In conclusion, a summary is provided of the main key parameters to be considered in the scaling transfer.

## 2. Scaling Effects in Organismal Design and Artefacts

At multiple levels of biological organization, living organisms can provide a wide range of functional solutions which include strategies related to physiology, morphology, dynamics, and behaviour, moving from molecular components to whole organisms and even populations (e.g., swarms) [21,22]. Although the scaling range is continuous and not discrete, a common and general distinction can be made between nano-, micro-, and macroscopic scales. In biology, a relative hierarchical size subdivision can be identified as follows: Molecule (nm), Cell (µm), Organ (mm/cm), Individual (cm/m), and Population (km) [23].

In nature, size influences organismal design and function. Size decreases or increases during ontogenesis (i.e., organismal development from egg fertilization to adulthood), as well as during species evolution, and its variation drastically affects the anatomical, physiological, and behavioural traits of organisms. The study of how these traits change with size is called “Allometry” [24]. Size and organismal design are strictly connected and changes in scale can lead to proportional or non-proportional variations of body traits [18,24,25]. Many scaling relationships can therefore be described through the following Equation:

$$\log\;y=\log\;a+b\log\;x\;\mathrm{or}\;y=ax^{b}$$

where x is a body size, y is an observable trait, a is a constant and b is the scaling exponent or *allometric coefficient* [3,5,14,24,25]. Considering different developmental stages, the allometric coefficient identifies the differential growth ratio between a body part and the entire body. Hence, when a body part has a higher growth rate than the entire body, the allometric coefficient is b>1, indicating a positive allometry (or hyperallometry), e.g., the large chela of the male fiddler crab (*Uca pugnax*) compared to its body, whereas when a body part has a lower growth rate than the entire body, the coefficient is

b<1

indicating a negative allometry (or hypoallometric), e.g., the human head that grows at a minor rate compared to the rest of the body and, subsequently, becomes proportionally smaller as the individual grows to adulthood [18,24,25,26]. Some other examples of variables obeying to this scaling law are metabolic rate, heart rate, DNA nucleotide substitution rate, lengths of aortas and genomes, tree height, density of mitochondria, and concentration of RNA [14,27].

In organisms, body mass is approximately proportional to the cube of the organismal linear size, while surface area varies proportionately to its square [3,5,28]. Therefore, the organismal surface area is roughly proportional to their body mass raised to the power of ⅔ [3,5,27,28]. This causes cellular diffusion problems for oxygen and food intake when the organismal size increases [3,5,27,28]. In multicellular organisms, these problems determine structural change in body shape and complexity, increasing the surface area, such as convolutions (e.g., human intestine and brain), intricate architectures (e.g., sponge shape variations in Asconid, Synconoid and Leuconoid) and/or the geneses of specialized devices (e.g., circulatory and digestive system) [29,30]. Another scale issue is strength, which drastically affects the shape of organisms in size scaling. Increases in size determine surges in body weight that need to be sustained. This is the reason why giant sequoias develop a larger and more solid base compared to smaller trees. In growing trees, it is also possible to observe drastic changes from thin branches and trunks to thick, massive, and resistant ones [28]. During growth, organisms must increase in size without interrupting their function and, because of the weight increase, an adult stage is not simply an enlarged version of a juvenile. The organism weight follows the cube of its height; thus, in an organism which doubles in length isometrically, its volume and mass will increase by a factor of eight (*Square–cube law*, that was first described by Galileo in his book *Two New Sciences*, 1638) [3,5]. Allometrically, the body changes its proportion during growth to sustain this physical demand [3,5,27,28].

Other researchers recognized that many physiological (e.g., metabolic rate, generation time, longevity), behavioural (e.g., running speed) and ecological (e.g., abundance) traits also scale with body size [3,5,14,28,31]. Body size changes are also visible during evolution from the first bacterial cells to multicellularity. Enormous sizes reached by plants and animals increased and decreased during geological times (for a review see [30]). The increase and decrease in size require adequate structures, synthesis of materials and other favourable morphodynamical factors [3,5,18,27,31,32]. One of the factors influencing the natural selection for size is the ecological principle of intra- and interspecific competition since each size level occupies specific ecological niches (i.e., the role an organism plays in the environment and its interaction with the biotic and abiotic factors) [31,32]. The increased size of some taxa could be correlated with a decreased predation, increased longevity, greater intelligence, or enhanced competitive abilities leading to a differential survival; however, it can also cause a reduction in reproductive output and/or population density and create longer generation times and greater overall resource requirements [31]. Interestingly, a specific pattern of body size evolution was also identified in insular habitats, acknowledged as the island rule, for which large taxa tended to become dwarfed, most likely due to the reduced resources availability (e.g., pygmy mammoth, elephant, hippopotamus, boa, sloth, and deer), whereas small taxa tended to become larger due to a reduction in predation pressure (e.g., birds, rodents, and Galapagos tortoises) [33].

Like organisms, artefact size is related to functions, expected forces and performances. In technology, a relative hierarchical size can be identified as follows: Nanotechnology (nm), Materials (µm), Products (mm/cm), Large Products (cm/m), and Distributed Systems (km) [23].

Computers, trains, airplanes, boats, building constructions and other manmade artefacts/technologies are drastically variable in size, and the working principles of these designs are totally different. In engineering, scaling can be obtained using dimensional analysis and “similarity theories”, which provide a method to understand system behaviour when scaling occurs [19,34]. Specific scaling laws in the form of mathematical equations and models were developed to provide answers to different biological questions, designs, and problems: e.g., strength-size relationships in porous materials of coconut endocarp and sea urchin spines [35], and the optimization of a crack propagation system or wind turbine rotors scaled to a desired size [19,36,37].

In solution transfer, biological information can be chosen at one scale level and applied to another. However, common and universal guidelines in scaling natural systems are presently far from being identified, since they highly depend on the biological working principles, as well as the specificity of the final application field. Notably, Vincent et al. [23] discussed in detail the differences between biology and technology with regard to six fields (substance, structure, energy, information, space, and time) and with sizes ranging from nanometres (atoms) to kilometres (town or ecosystem) based on TRIZ database. TRIZ, the acronym of *Teorija Reshenija Izobretatel’skih Zadach* or the “Theory of Inventive Problem Solving”, is a collection of tools and techniques for the successful transfer of inventions and solutions from one field of engineering to another, which are also successfully used in biomimetics [1,23,38]. The correlations developed by Vincent et al. [23] led to important evaluations in technology, particularly regarding the vast amount of energy (60% of the time) used to develop diverse materials with novel properties. In contrast, biology invests the minimum energy (5% of the time), using few materials and synthetic processes, where energy contribution is high, and more structural organization, e.g., hierarchy, where energy is negligible. In this way, Vincent et al. [23] provided an important biomimetic lesson: “instead of developing new materials each time we want new functionality, we should be adapting the materials we already have”. In this regard, scaling analogies and differences between organisms and artefacts can encourage cutting-edge, sustainable and biologically inspired designs increasingly close to the construction law of organisms. Currently, this is also supported and enhanced by the increased instrumental resolution and virtual investigations allowing for the effective reconstruction and analysis of the organismal design functional traits, as well as the contemporary technological innovations pursued in digital productive process (e.g., additive manufacturing) generating new opportunities for mimicking complex multiscale, multi-material and multifunctional structures at different scales [39,40]. In particular, the 2D/3D reconstruction of natural structures can be obtained using different techniques, mainly based on image acquisition (e.g., photogrammetry), laser scanning or more detailed computational tomography (in micro- and nanotomography, the pixel sizes of the cross-section are measured in micro and nanometres) [41,42]. Additionally, different numerical analyses allow for the study of these complex biological structures, such as finite element analysis that can effectively investigate the performance of 2D/3D biological models providing insights on the morphology, function, and evolution of extinct and existent species [43]. Finally, the 3D modeling, experimental–numerical simulation and digital fabrication can, nowadays, provide new ways to analyze and recreate complex functional structures of the biological field from the macro- to the nanoscale, leading to novel, inspired technologies and artefacts. These include additive manufacturing with its different technologies (over 50), which are demonstrated as an effective method to prototype and manufacture products with a good control of the resulting geometric shapes; thus, an appropriate tool to address the fabrication challenges of complex biomimetic materials and structures [39,40]. Technologies such as 3D laser lithography systems (e.g., Nanoscribe) provided a fast and powerful tool for micro- and nanofabrication that revolutionized the field of bioinspiration and biomimetics. These tools allowed for the recreation of micro/nano patterns and textures of organisms to realize innovative soft materials and functional surfaces (e.g., artificial surfaces with three-dimensional and hierarchical micro-elements able to retain gas inspired by the Salvinia leaves hairs) [44,45,46,47].

In the following paragraphs, an overview regarding organismal design and a catalogue of bioinspired solutions is provided with regard to scale-related principles.

### 2.1. Mechanics

#### 2.1.1. Statics

A long history of research focused on the relationship of organismal materials and structures together with their mechanical functions [3,5,48,49,50]. One of the main functions of structural materials, elements and systems in organismal design is mechanical support and protection [3,5,49,50]. Mechanical function can be investigated by applying notions and principles of structural mechanics to organisms by examining their material properties and behaviour at different hierarchical dimensional levels, from the nano- to the macroscale. This approach is defined as biomechanics and can lead to the identification of numerous mechanical–structural principles, based on the physical–mathematical laws that govern the structure/function relationship in organisms [3,5,49,50]. On par with man-made structures, organisms need to resist the biotic and abiotic stresses that they are subjected to in their environments [3,5,49,50]. Mechanical performances are influenced by size. Many aspects have exponential relationships with morphological features, e.g., the deflection of a bending beam bearing a load is proportional to its length. Particular functions, such as skeletal support and visceral protection, are generally insensitive to structural variation at a small size, but extremely sensitive to morphological changes at a large size [3,5,27,48]. For example, Kingsolver and Koehl [51] demonstrated how the function of similar structures in the flying appendages of arthropods changes remarkably according to its change in size.

From the nano- to the macroscale, biological structures inspired functional strategies, particularly *apropos* material science, industrial products and building constructions [2,23,39,40,52]. In material science, natural materials are studied in detail due to their ability to manage structural forces and protect from damage caused by static and dynamic mechanical loads using adaptability, flexibility, hierarchic layers, self-organization and repair [39,40,53]. The current analysis and modelling tools reveal that their unique properties rely on their complex structures ranging from the nano to the macroscale. For example, nacre has a “brick and mortar” layered architecture, consisting of aligned crystalline aragonite platelets (bricks 0.5 µm thick and 8–10 µm wide) surrounded by polymers composed of chitin and proteins (mortars 20–50 nm thick) [54]. This structure provides unique strengthening and toughening mechanism properties, preventing crack propagation, and allowing energy dissipation [54]. At a similar scale, Wang et al. [55] realized an artificial nacre of alumina microplatelets and graphene oxide nanosheets-poly (vinyl alcohol) (Al_2_O_3_/GO-PVA) through a layer-by-layer bottom-up assembly, which exhibited a strength (143 ± 13 MPa) and toughness (9.2 ± 2.7 MJ/m^3^) considerably superior to the natural nacre (80–135 MPa, 1.8 MJ/m^3^). Another example was the butterfly wing nanostructures that were efficiently reproduced using two-beam super-resolution lithography, leading to high-performance artificial nanostructures with an elastic Young’s modulus enhanced by 20% [39].

At the microscale, numerous cellular and lattice structures characterized by a high strength-to-weight ratio are present in nature, such as wood cell walls and bone trabecular systems [56]. These microstructures are successfully reproduced at the same scale using an *Aerosol Jet 3D* printer to achieve new material lattices. This printing technique deposits aerosolized microdroplets comprising metal nanoparticles that allow the assemblage of nanoparticles in an intricate microscale 3D network [57]. The final products are bioinspired micro- and nanolattices, which are very strong and lightweight due to their high porosity [39,57]; applications for these materials are found today in tissue engineering and energy storage, as well as microfluidic, microelectronic, and optoelectronic devices [57].

A macroscopic scaling-up (ten times) was carried out by Song et al. [58], who considered the high flexibility and impact resistance of fish scales in the three-spined stickleback (*Gasterosteus aculeatus*) to create an inspired 3D-printed armour used with multiple degrees of freedom for protection. The prototypes were fabricated based on CAD models obtained from a microcomputed tomography scan of the stickleback. The fish scale porose replicas were beneficial for bending stiffness and strength at a minimum weight, enabling multiple translational and rotational degrees of freedom [39].

Microstructural geometries of planktonic organisms, such as diatoms, radiolaria, tintinnids, acantharia, and foraminifera were investigated for their potential mechanical effects and macroscopically scaled-up as lightweight structures, e.g., a bicycle, helmet, and an off-shore foundation [59,60,61]. Indeed, a scaling transfer of lightweight and resistant structures to technical product solutions is generally possible, considering the basic physical aspects. In technical load cases, the materials and fabrication processes differ considerably from those of organisms. Hence, suitable technical adaptations are usually necessary. In this regard, interesting successful examples include the transfer of microscopic siliceous valve geometries of diatoms and other planktonic microorganisms in the *ELiSE Lightweight Process* technology, resulting in a range of lightweight and resistant products [60].

Building constructions, biological morphologies, structural strategies, and generative processes inspire shapes, structures, and fabrication processes. Many living organisms employ protective shells against predators and environmental forces, and some of them, such as *Acanthocardia*, *Pecten* or *Tridacna* seashells, use corrugation as strategy to optimize structural performance, increasing the structural resistance of shells, reducing thickness and, consequently, the amount of material employed [2,62]. The shell corrugation strategy is successfully employed in building constructions to increase shell stiffness and strength against variations of loading conditions, with a limited increase in structural thickness and weight (saving materials, and thus economic and environmental advantages). The aircraft hangar at Orly Airport, designed by Buckminster Fuller and completed in 1923, is an exemplary masterpiece. It consists of a reinforced concrete structure constituted by a folded paraboloid shell covering a space of 61 to 91.5 m^2^ [63]. A similar strategy in nature is the lamellar structure of fungi digitally recreated by the Almond Studio team and applied in a line of interior design products, called *lamella,* printed in 3D and providing sustainable alternatives to shelves, chairs and tables in a well-optimized and effective way [6].

**Figure 1:**
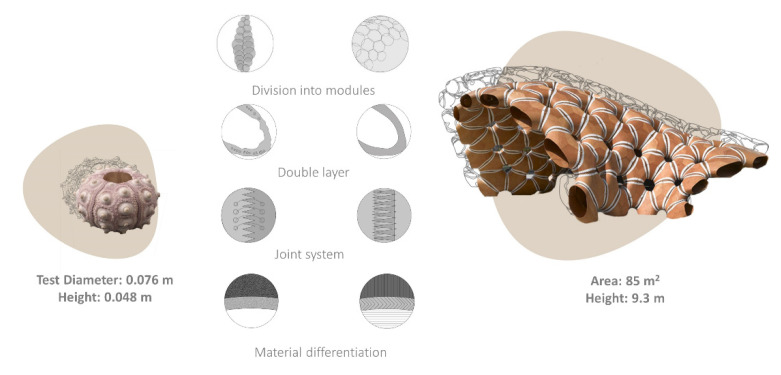
Scaling in Architecture. ICD/ITKE Research Pavilion (2015–2016) inspired by morphological and mechanical principles of the genera *Clypeaster* and *Phyllacantus*. The following details were scaled: (1) division into modules; (2) double layer modules, (3), modules interconnected by finger-joints and collagen fibres (4) and material differentiation. Pavilion image adapted from [67].

Another constructive strategy is shell segmentation with flexible sutural ligaments. This solution can be observed in organisms, such as the turtles and sea urchin skeletons, which inspired new building constructions and industrial design products [64,65,66]. ICD/ITKE Research Pavilions and permanent buildings are outstanding examples of architecture inspired by echinoid skeletal structures [64,65,66]. The Stuttgart pavilion (2015–2016) effectively demonstrates the scaling of different structural echinoid details: (1) division into modules, (2) material differentiation, (3) double layer modules, and (4) modules interconnected by finger-joints and collagen fibres (Figure 1).

Charpentier and Adriaenssens [68] carried out an interesting study on thin shell structures from biology to engineering and across their different scales to establish their scalability limits. They considered 64 thin shell structures related to five categories: (1) engineering stiff and (2) compliance, (3) plant compliance, (4) avian egg stiff, and (5) microscale compliant shells. The main parameters used to describe and analyse the scale of these shells and their mechanics were: dimension, thickness, material, Poisson’s ratio, Young’s modulus, volumetric mass, and the influence of gravity (determining at what scale gravity becomes relevant enough that the bending deformation in a shell related to self-weight becomes larger or smaller than the initial size of the shell). The results demonstrated that all the considered shells exhibited a similar mechanical behaviour across the different scales. Thus, microscaled biological shell geometries can be generally upscaled to macroscaled engineered shell geometries; however, compliant thin shells are identified as prone to self-weight deformation at a large scale due to the effect of gravity; therefore, its applicability depends on the nature and needs of the final application (i.e., if this mechanical behaviour compromises the working principle).

Regarding the mechanic realm and scaling properties, auxetic structures need to be considered. These structures are characterized by a negative Poisson’s ratio; thus, if stretched, they increase in volume perpendicularly to the applied force [69]. Auxetic structures are observed in nature, e.g., aquatic salamanders, cow teat skin, cat skin and snakeskin, providing important functions such as effective mechanical behaviour, flexibility, adaptability, and dynamicity [70,71,72,73,74]. Depending on the constructional geometry, auxetic behaviours can effectively be applied at different dimensional scales. In this regard, the advancement of additive manufacturing also improved the applicability and versatility for their production [64,75]. Bioinspired auxetic structures led to a new line of materials for textiles, aerospace, biomedical, sensors and actuators (for a review see [76]), as well as extremely lightweight, adaptable, and dynamic design products such as seats, cervical collars, bags, etc. [77,78].

#### 2.1.2. Dynamics

Dynamics is the study of systems in motion. Living organisms, especially vagile animals (i.e., walking, swimming, and flying animals), adapt their structure and movement to terrestrial, aquatic, and aerial environments increasing their efficiency with minimum energy. Organisms use locomotion to escape predators, search for food, mate, or search for new habitats. Hence, during evolution, different anatomical adaptations, e.g., flexible muscles and complex architectural structures, and behaviour traits were selected [50]. Recent technological advances in computational analysis led to a new precise evaluation of these systems, building models that mimicke these biological designs, especially in robotics. Indeed, numerous examples of robots mimicking organismal shapes and movements emerged in recent decades, including air [79,80], underwater [81,82], and ground robots [83]. Notorious biological translations are the robotics arm OCTARM, inspired by the octopus [84], the Elephant Trunk Manipulator [85], and the numerous demonstrators developed by Festo Bionic Learning Network (Bionic Swift, BioincWheelBot, BioincANTS). By mimicking animal motions, these macro- and microrobots are characterized by complex movements with a remarkable improvement in their locomotion, mobility, grasping, and recognition systems, performing tasks impossible for humans to perform. Accordingly, the field of biomimetic/bionic robotics is in great expansion and prefigures new and wide opportunities for applications at both a micro- and macroscale, such as space exploration, healthcare, industrial automation, logistics and agriculture [86]. The biological transfer in robotics mainly occurs at the same scale to obtain similar performances in similar environments; however, different scaling transfers are reported. Of course, the application of bioinspired dynamics and related adaptive strategies is not only limited to robotics, but also extended to materials, aerospace, automotive, textiles, energy production, building constructions and many other industrial sectors. Some of the most interesting technologies are those stretchable and adaptive materials that imitate the ability of living organisms to change their shape reversibly and dynamically for camouflage, locomotion and grasping [87]. For example, synthetic tissue groupings inspired by cephalopod skin were developed. They consisted of a fibre concentric mesh embedded in a silicon elastomer and had the ability to be programmed to transform from 2D planar surfaces into complex and predefined 3D hierarchical shapes, e.g., natural stone and plant, and to camouflage with background environments [88].

##### Walking, Jumping, and Running

As the size and weight of animal species increases (from fleas to frogs, and then rabbits, dogs, leopards, and elephants), the body structure changes as well as its form of locomotion, ranging from walking to running and jumping [50].

In robotics, scale effects on motion are analogous to organismal adaptive challenges. By studying the scale effect mechanism in animals, Liu et al. [89] analysed the influence of a hexapod robot mass and the characteristic size on its feature locomotion. They revealed that: (1) the foot force rises when robot mass increases, (2) the foot force of the unit robot mass decreases when the mass increases, (3) the maximum joint torque increases when robot mass increases, (4) the system power increases when robot mass increases, (5) the system power of unit of robot mass is constant, (6) the peak system power decreases when the distance between the front and rear leg increases, and (7) the joint torque increases when the distance between the legs increases.

As the robot decreases in size, the obstacles in the environment become greater compared to the larger robot (the “Size Grain Hypothesis”) [90]. Bionic hopping movement was studied in recent years to improve the robot’s ability to adapt to unstructured environments, avoid dangers and overleap obstacles [91,92]. Studying the locust morphology and systems, Kovac et al. [91] developed a hopping robot with a jumping mechanism that overcame obstacles of more than 27 times its own size (5 cm). The hopping mechanism was also studied and applied in large ground robots. Taking inspiration from a biomechanical study on a vertebrate musculoskeletal system, Niiyama et al. [93] constructed ‘Mowgli’, an electro-pneumatically actuated bipedal robot able to jump at a height over 50% its size (3 Kg weight, 0.9 m height) and land softly. Graichen et al. [94], described ‘BionicKangaroo design’, a bionic demonstrator (7 kg weight and 1 m height), developed by Festo Bionic Learning Network, which resembles the unique hopping movements of a kangaroo, and can jump up to 0.4 m of its height, covering a distance of 0.8 m.

In material science, there is a great interest in the development of soft and responsive materials capable of producing the mechanical work necessary to activate autonomous motion. In this regard, the muscles of mammals, able to contract and relax in response to nervous signals, were studied by Li et al. [95], leading to a light responsive soft material made of nanoscale peptide assemblies and polymer networks, activated by blue light. In this case, the transfer of scale is very difficult to identify and mimic, since the dynamism is macroscopic, but the process occurs at a micro- and nanoscale, guided by a neural and physiological response.

##### Swimming

Size drastically affects a moving body due to the Reynolds number (R*e*), defined as the ratio between the inertial and viscous forces [3]. R*e* drastically affects flying and swimming organisms. Large organisms, such as whales, have a high inertial force, and thus a large R*e* (200,000,000), which facilitates their motion, whereas small organisms, such as bacteria, have a low R*e* (0.0001) and swimming can be very difficult [3,28]. The organismal design in the diverse scale realms is significantly different. Locomotion at very low R*e* is usually obtained using appendages, cilia, or flagella. Larger microbes adopt relatively huge and complex systems of cilia or flagella, whereas bacteria, which have an even lower R*e*, have simple flagella attached to a rotor producing a rotating movement similar to that of wheels [96].

Nektonic organisms, such as fish and cetaceans, adapt their body shapes and surfaces to improve swimming performances mainly through a drag reduction and an increase in propulsive forces [97]. Drag is the hydrodynamic force representing the resistance of a body in a fluid environment. Therefore, it is the force that dislocates sessile organisms and prevents swimming, flying, and sinking motions [48]. In aquatic organisms, the primary strategies of drag reduction are the body streamline profiles which inspire robots of similar scale, as well as large scale designs, e.g., the USS submarine hulls, *Albacore* (AGSS-569) (1953) [98].

Osteichthyan and Chondrichthyan fishes have unique dermic scales that provide protection and biofouling repellence and reduce frictional fluid drag during swimming [99]. Riblets mimicking the scale patterns of fast-swimming sharks reduce drag [100]. Recently, using microcomputed-tomography (micro-CT) imaging and 3D printing, shark dermal scales were shown to significantly increase swimming speed (up to 6.6%) and reduce static drag (up to 8.7%) compared to smooth surface [101]. A variety of sharkskin-inspired materials were produced to reduce the drag of submerged bodies and notoriously applied in the *Speedo Fastskin* swimsuit [102]. Material scientist A.B. Brennan discovered that sharkskin-like textures inhibited biofouling and bacterial growth with respect to smooth surfaces and created a new material called ‘Sharklet’ [103]. This finding was applied to reproduce the bottom covers of boats, as well as in medical devices and on hospital surfaces [104]. In this case, although the application scale is very different, the artificial scale pattern is the same as the sharkskin, different functions would not be possible.

**Figure 2:**
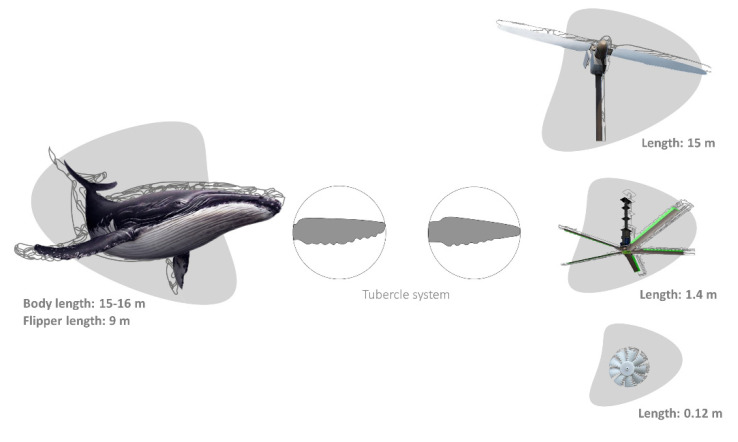
Biomimetic scaling of the tubercle effect. Tubercle system of humpback whale fin provided several bio-inspired designs at different dimensional scales: Wind turbine blade (15 m), indoor ventilation fans (1.40 m) and fans (12 cm) developed by the Whalepower Corporation. Images adapted from [111].

Similarly, other biomimetic potentialities focus on the propulsive systems of aquatic organisms, mainly on their fin morphology and flexibility, as well as the oscillatory body motions of their bodies [98,105,106]. Large cetaceans, such as whales, developed unique flippers with tubercles located at the leading edges capable of a high manoeuvrability and a unique hydrodynamic performance to execute tight turns and efficient swimming, an imperative task for feeding [107,108]. The tubercle function was described in the humpback whale, *Megaptera novaeangliae* by Borowski, 1781. The humpback whale has the longest flipper of any cetacean, varying from 0.25 to 0.33 of its total body length [109]. The tubercles can maintain lift, prevent stalling, and decrease the drag coefficient during turning manoeuvres [108]. This inspired several new bio-inspired designs, e.g., surf fins, watercraft, aircraft, ventilation fans, and windmills developed by the Whalepower Corporation [107,108,110] (Figure 2). Regarding the scale effects, tubercles and flippers work at the same size and Reynolds regime of many technologies, leading to a large range of engineered applications in air systems [107,108]. In the case of flying vehicles, particularly limited dimensioned ones, the importance of edge vortices became significant, leading to an increased localized turbulence, as well as to the possible occurrence of stalling due to insufficient lift. This could be avoided through the application of serrated wing edges. A proper and hierarchical saw-toothed profile based on tubercles constitute wing edge defects (also applicable to wind turbines) that can increase the lift and the payload in a more consistent way [107,108].

##### Flying

Flight is strongly dependent on the size and relative R*e* that control the organismal design, flight mechanisms and their aerodynamic performance. Among the size-dependent issues, staying aloft and moving forward are the main ones: the first is obtained through an upward force able to counterbalance the weight that depends on volume; the second necessitates a force equal to the drag generated by the flight speed, which depends on the surface area [3,5]. Small flying insects can easily stay aloft against the gravitational pull but have greater problems in moving forward than birds and airplanes [3,5,50]. Flying animals inspired humans for centuries, paving the way for aeroplanes, and still inspire flying technology today. However, the main issues remain in the scaling of structures and working principles that organisms and technologies use. Artificial systems are usually large in size and fast in speed compared to organisms that are small and relatively slow. For example, aircrafts carry greater loads and fly faster and at higher altitudes than birds [107]. Additionally, the stresses generated by high-speed flight do not allow a direct replication of the variable organismal kinematics, aerodynamics and morphologies used to produce complex flight performances and successful turning manoeuvres [107,112,113]. These differences in scale strictly limit a direct translation of organismal designs to human technologies; however, effective bioinspired strategies have brought significant innovation in different sectors, particularly in the aerospace and robotic sectors.

In the late 1970s, R.T. Whitcomb, a NASA Langley Research Center engineer, was inspired by the flight characteristics of soaring birds and their tip feathers that maximized lift with a minimum wing length, developing the concept of “winglets” that was successfully applied to different large-scale aircrafts reducing lift-induced drag and improving flight performance [114]. Organismal flight, especially in flapping flying animals, significantly inspired robotics and drones of different dimensional scales. An active research area is dedicated to the development of micro air vehicles (MAV), i.e., insect and bird-size drones who perform autonomous flight are useful for environmental monitoring and surveillance. MAVs have a maximal dimension of 15 cm, flight speeds of about 10 m s^−1^ and a function in the Reynolds number regimes of most flying animals (10^−4^–10^−5^). In their review, Liu et al. [113] described in detail the biomechanics and biomimetics of biologically inspired flight systems from the insect kinematics, aerodynamics, wing morphologies and the relationship with the Reynolds number. Since aerodynamic and energy conversion efficiency decreases with scale, these small aerial robots are usually constrained to short mission times and a good solution is perching them as biological flyers. In this regard, Roderick et al. [115] provided a complete state-of-the-art design concerning the perching performance of aerial robots, as well as a unique overview of the broad range of underused solutions that animals demonstrated for perching on surfaces in their environment. They identified the main scaling issues, describing how the maximal size of perching animals and robots is limited by scaling laws both for the adhesion and claw-based surface attachment.

##### Tropic, Nastic, and Other Movements

In nature, dynamic systems are present even in sessile organisms, which constantly need to respond and use external environmental agents to optimize their survival, growth, and reproduction. Plants evolved unique reversible or irreversible movements categorized into two main groups: (1) tropic movement, dependent on the direction of the stimulus and associated with growth tissues; (2) nastic movement, independent from the direction of the stimulus and associated with either a growth process or change in turgor [116]. The stimuli determining tropic movements are gravity, water, light, and touch, whereas stimuli such as temperature, humidity, touch, and light exposure result in nastic movement [117]. Other types of strategies can be found in the dispersal mechanisms of their seeds using mainly animals, wind, and water. In this regard, plants adapted strategies that considered the relationship between mass, structure size, and morphology. For example, in the wind dispersal of the Asteracea, the plant used a pappus, i.e., modified calix acting as a hairy parachute, which was efficiently scaled on the basis of the attached seed size [118].

Many of these plant movements and dispersal systems are based on macrolevel changes in shape based on the hierarchical actions occurring at the meso- and microscale of their materials and structures [119,120]. A classic example is the Venus flytrap (*Dionaea muscipula*) and its modified leaves. The leaf motion is achieved at a macroscale when the three trigger hairs (mesoscale) located on the epidermis are stimulated. Stretched in an opening position storing potential mechanical energy, the leaves undergo the action potentials and osmosis processes of their membranes (microscale), which release stored energy and change their shape, determining the rapid closure of the trap [121]. Further examples are pinecones, which macroscopically change their shape based on the hygroscopic expansion of its cells (microscale), according to the degree of humidity ensuring seed dispersion in favourable environmental conditions [122]. These shape-changing properties of plant systems were studied in detail and inspired the development of artificial structures, materials, and systems able to respond to the environmental changing conditions (e.g., humidity, heat, and light) [39,123,124,125]. They are extensively scaled and applied in architecture, since buildings share similarities with plants due to their static nature and need to regulate their temperature and access to light [123,124,125]. Hence, unique façade systems inspired by plant movements have been developed. In this regard, a bioinspired example is the Flectofin^®^, a façade-shading system inspired by the Bird-of-Paradise, *Strelitzia reginae*. The modified leaves of this flower present a bending mechanism when external mechanical forces are applied, providing a support site for pollinating birds. This mechanism is replicated using glass fibre-reinforced composites with effective elastic deformation applied as a hinge-less façade-shading system [126]. The moisture-driven movement observed in spruce cones attracted a great deal of attention in architecture. Generated by a passive response to humidity alterations, this movement does not re-quire a sensory system or motor function but depends on the material hygroscopic behaviour and its own anisotropic characteristics. The design of the HygroSkin mete-orosensitive Pavilion, commissioned by the FRAC Centre in Orleans, France, was conceived from the biomimetic investigation of the spruce cone principles. It was constructed with a robotic fabrication using 28 components with humidity-responsive apertures based on thin planar plywood sheets responding to humidity changes within a range from 30% to 90% [127].

Plant movements have inspired different robotic solutions [128]. Tropic plant roots can move within the substrate along the gravity vector and towards water and nutrients [116]. This movement inspired the work of Mazzolai et al. [129] in the development of a miniaturized mechatronic system activated by hydraulic pumps named Apex (62 mm of length and 22 mm of diameter). It consisted of different sensors, detectors and controllers with a bio-inspired algorithm reproducing gravitropism (i.e., tropism simulated by gravity) and hydrotropism (i.e., tropism simulated by water) behaviour, as well as a bio-inspired osmotic actuator module of three cells separated by couples of osmotic and ion-selective membranes [129]. Further developments led to PLANTOIDS, robotic systems inspired by plant roots for soil exploration and monitoring [128]. In this field, other examples are works by Tonazzini et al. [130] nd Mishra et al. [131] for the study and development of robotic probes with root-like morphologies able to better accomplish penetration tasks. In particular, the latter working group developed an artificial root-like shaped probe inspired by *Zea mays* roots using 3D printing technology, which is more efficient in terms of penetration force and energy consumption compared to standard shape probes [131].

The dispersal strategy of tumbleweeds has been scaled and applied by the NASA Jet Propulsion Laboratory (JPL) in the creation of a spheric robot with a diameter of 1 m [132]. Whereas the unique flight of rotary-winged seeds is applied in *Samara*, a hovering monocopter (15 cm in size) with an active control of the feathering angle is able to generate a lateral motion [133].

In material science, different smart fabrics have been inspired by plant stoma (or stomata) [134]. They are gas-exchange openings present in the lower epidermis cuticle of leaves and regulated by two specialized cells (guard cells) according to humidity [135]. An inspired system was replicated using a dome-shaped pattern in neoprene with centre hole and commercialized by *Stomatex* as a heat acclimation fabric for sports and orthopaedic supports [136]. Similarly, adaptive textiles were also inspired by pinecones and activated by moisture concentration. Dawson et al. [122] demonstrated the pinecone opening system. Later, this bioinspired mechanism was applied to a textile prototype, composed of a woven polyester fabric on one side, coupled with a non-porous, hydrophilic polyether-ester block copolymer membrane, characterized by a “U”-shaped perforation, positioned across the bilayer fabric, which opened and closed as the moisture level increased and decreased [137]. These opening and closing movements can be scaled by appropriately designing thicknesses, morphology, and the relationship between the shrinking and widening of the material induced by water absorption. There is also a growing interest in the development of bioinspired materials for the reversible attachment to different surfaces. Plants have inspired different mechanical attachment strategies, such as the famous hook-based interlocking of zoochory seed dispersion, which inspired Velcro. Similar attachment devices have also been inspired by climbing plants, a recent example provided by Fiorello et al. [138]. Inspired by the microscopic hook mechanism of *Galium aparine* leaves, they successfully realized a flexible mechanical interlocker characterized by 3D-patterned hooks of the same microscale using multiphoton absorption laser lithography, ensuring the high replicability and scalability for the technical application in soft- and micro-robotics, the textile industry, and biomedical fields.

### 2.2. Optics

Optics entails the behaviour and properties of light. In nature, living systems are mainly based on light exploited as energy and food, as well as used to communicate and perceive the world and its diversity [139]. In different living kingdoms, the ability to perceive, use and manipulate electromagnetic radiations provides many advantages in feeding, reproduction, and defence. Accordingly, a variety of powerful colours and functional patterns characterize organismal designs, profiting from light ranging from infrared (IR) to visible (VIS) (i.e., wavelengths from about 380 to about 750 nanometres) and ultraviolet (UV), with different degrees of polarization: e.g., giraffe, zebra, jaguar pigmentation patterns [140]; hummingbirds, beetles and butterfly structural colours [141]; the bright UV-patches of the blue-moon butterfly and the peacock mantis shrimp [142,143]. In this regard, colours in nature have three main sources: (1) pigments, inducing the selective absorption of light, transmitting and reflecting the other parts of the electromagnetic spectrum; (2) structural colours, created by the selective reflection or coherent/incoherent scattering of the incident light impinging on highly structured and unstructured multilayer or hierarchical architectures; and (3) bioluminescence, the production and emission of light as a result of biochemical reactions using light-emitting molecules and enzymes [141,144,145]. Each source utilizes principles that function at different scales (especially at a nano- and microscale) and are mainly based on intra- and interspecific perceptions. Indeed, powerful colours are primarily used for: intraspecific communication, attracting mates or exchanging other intraspecific information (e.g., intrasexual competition, territoriality); interspecific communication, attracting other organisms (e.g., flowering plants and pollinators); sending clear signal warnings to potential predators on their poisonousness (aposematism); or not sending any signal-assuming colour, form and behaviour of the surrounding environment (camouflage) [146,147,148]. Presently, many of these organismal optical strategies are applied at a nano- and microscale, e.g., nanotechnology and new materials, to a macroscale, e.g., airplanes and ships. Technological advances have led to the invention of new materials (i.e., metamaterials) and methods allowing light manipulation, even at the nanoscale, in turn influencing the range of optical products that can be designed [145,149,150]. Light control and processing play a key role in telecommunications, imaging, sensing, biomedicine, energy harvesting and many other fields [151]. In optics, scale factors reveal significant constraints.

#### 2.2.1. Pigmentary Colours

**Figure 3:**
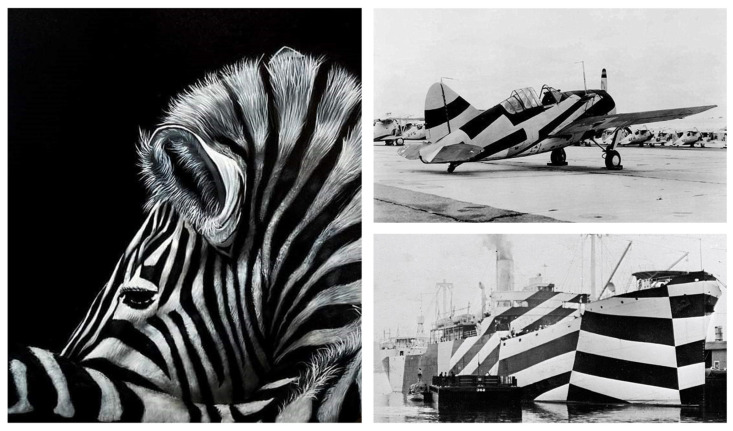
Dazzle patterns of zebra. U.S. Navy SS West Mahomet ship, 1918, and Brewster F2A Buffalo fighter aircraft, 1937 (Zebra image: oil painting of the artist, Ciro Ciardiello; ship and aircraft images retrieved from [159]).

Biological pigments, or biochromes, are commonly found in algae, plants, and animals. Their functions can be either biochemical and metabolic (e.g., photosynthesis) or biophysical and physiological (visual effect function and perception) [152]. Functional patterns, based on pigment distribution and its visual effect, have been mimicked leading to interesting applications. The camouflage effect of background matching and visual disruption has attracted major attention, especially for military applications. Smart materials and fabrics with adaptive camouflage systems have been developed to mimic the ability of cephalopods to rapidly change their pattern [153]. Disruptive colouration is another camouflage tactic in which the identity and location of a species is disguised through a specific colouration pattern [154]. The dazzle patterns of zebras, giraffes and jaguars have the ability to confuse observing predators or prey by misleading them using the shape, location, or movement of the animals in motion [155,156,157]. Historically, these patterns have been extensively used to protect battleships and aircraft in World War I and, to a lesser extent, from World War II onwards. Large-scale, high-contrast, irregular patterns have been painted on military navy and flying corps; however, the functional success and effective working principles of these have been difficult to verify [157,158] (Figure 3).

#### 2.2.2. Structural Colours

The presence of ordered optical structures in several animals, plants and protists is frequent and documented by observations of transmission and scanning electron microscopies. These structures can range from the nanoscale to the macroscale and are not necessarily related to the presence of pigments or materials with specific absorption in the visible spectral range. They were developed by organisms to reflect, absorb, and manipulate light [39,160,161] and examples of their successful technical applications are numerous.

The simplest photonic nanostructure in biological systems was detected in iridoviruses [162]. These viruses self-assemble in paracrystalline arrays within the cells of infected organisms, causing optical iridescence arising from multiple Bragg scattering [145]. Biological nanostructures, characterized by multilayer arrangements, acting as selective reflectors are quite diffused in several floral or faunal species. These biostructures determine the structural colours, which do not depend on pigments but rather on their geometrical pattern and refractive index contrast, with respect to the surrounding environment [145,163]. Moreover, the combination of multilayer interference with coherent scattering and/or diffraction can contribute to structural colouration and iridescence [164]. For example, the different colours (i.e., green, orange and purple) of the Japanese jewel beetle, *Chrysochroa fulgidissima*, are determined by interference from alternating layers of chitin and melanin, whose spacing varies in different locations [165]. The originating structural colours in the weevil, *Lamprocyphus augustus* (Marshall, 1922), are even more complex. In this insect, every scale of the exoskeleton is composed of nanostructured domains of ordered air holes within the cuticular matrix. These domains are composed by the same crystal lattice but are orientated differently, determining an angle-independent colouration [166]. One of the most well-known examples of structural colouration in nature occurs in several species of *Morpho* butterfly [167,168,169]. The upper side of these invertebrate wings is characterized by a brilliant iridescent blue colour determined by the presence of multiple layers of scales (200 nm–40µm) and their different angular orientation [170]. This intense structural colour strategy is successfully mimicked in textiles by designing a multilayer structure with different thicknesses (polyester-nylon) capable of producing coloured dresses that do not contain any pigments or dyes, saving energy and reducing water pollution [171]. Other applications regard functional coatings, unmatchable colour security coding, solar cells, selective gas sensors, highly selective and sensitive chemical sensors, and high-speed infrared imaging devices [172]. The technologies used to recreate these structures are numerous, involving top-down and bottom-up approaches, allowing bioinspired optical structures to be effectively fabricated at both the nano- and microscale with complex shapes for designed optical systems [173]. Zhang and Chen [172] mimicked the *Morpho* wing colouration, fabricating lamellae layers using a process based on electron beam lithography and the alternate development/dissolution on the PMMA/LOR superlattice multilayers, recreating different types of blue and green colours by varying the nanometric dimensions of the ridge grating period, layer thickness, branch and pillar widths.

In contrast to pigmentary colours, the scalability of structural colours is limited since the visual effect strictly depends on the physical interaction between the light waves and surfaces of these structures. In the visible spectrum, diffracting structures should be on the micron/submicron scale for light interaction [173]. In the project “Photonic and micro mechanic proprieties of diatoms”, funded by the Italian FIRB program, the focusing properties of a single valve of the *Arachnoidiscus* genus diatom were investigated using a multidisciplinary science/design approach. Diatoms are ubiquitous microalgae, whose nanopatterned frustules are known to protect microalgae from ultraviolet radiation and to focus the photosynthetically active radiation on their active centres [174]. The physics team demonstrated that the presence of the valve brings a significant contribution to the Optical Eigenmodes (OEi)-induced light squeezing and improves its performance [175].

Focusing and wavelength selection optical phenomena are, in general, scalable, in the sense that the principles and ideas working in micrometric structures at the visible wavelength (viz., organismal structures whose dimensional features are comparable with the wavelengths of the visible spectrum) can be translated in artificial macroscopic structures (for instance, antennas) manipulating microwaves and radio waves, i.e., electromagnetic waves whose wavelengths range from centimetres to several metres.

#### 2.2.3. Bioluminescence

Different taxa have independently evolved bioluminescent properties, including bacteria, fungi, fish, jellyfish, and fireflies [176]. Researchers carried out numerous studies to understand in detail the organismal bioluminescence processes, and their imitation and reproposing in human technology was the target of many scientific fields: from bioluminescence imagining and biosensors in genetic and biomedical engineering, to new lighting systems in industrial design [125,177,178]. Being based on biochemical reactions, the biomimetic transfer of bioluminescence has proven difficult to recreate and is frequently mimicked using other physical–chemical principles. For example, in textiles, the firefly bioluminescence inspired the design of glow-in-the-dark clothes using a large scale of light-emitting devices with fabric-printed circuit boards (PCBs) or coloured light-emitting diodes (LEDs) [179]. In this regard, the most promising field for the technological use of bioluminescence is synthetic biology, able to directly re-design the existing biological systems for useful applications [178].

### 2.3. Electricity

Different living organisms have evolved the ability to communicate, perceive, protect, and feed using electricity. From small bacteria to large mammals, electricity is independently developed in multiple ways. Particularly, electric pulses in organisms can be generated (electrogenesis) and/or perceived (electroreception) [180].

Some genera of bacteria are discovered to generate and use electricity, e.g., *Shewanella* and *Geobacter* [181,182,183]. These bacteria have adapted to low-oxygen environments, directly using electricity as electrons harvested from rocks and metals, as a form of survival energy [181,182,183]. This ability is also exploited in the development of microbial fuel cells (i.e., devices for the current generation employing bacteria) [181].

On the other hand, complex multicellular organisms, such as vertebrates, obtain their electrons by oxidizing carbon from food through a series of chemical reactions within their cells that release electrons, thus reducing the oxygen obtained by the respiratory system. Indeed, all cells rely on redox reactions for energy production, in which electrons are released from a donor (oxidation) and are received by an acceptor (reduction). This flow of electrons provides power to the organismal bodies; therefore, whether the organism is a single-celled bacterium or a huge blue whale, the ultimate challenge is to find a source of electrons [182,183]: the difference lies in their scale. As reported in Section 2, the increase in the multicellular organism scale determines an increased body complexity and the necessity of specialized systems and processes able to sustain a multitude of cells.

Beyond the energy production for sustaining life, sophisticated strategies to use electricity can be identified in the animal kingdom, notably in fish [180]. Various species of freshwater and saltwater fish (e.g., electric eel, African freshwater catfish, elephant-nose fish and the torpedo ray) have functional anatomical structures able to generate up to 860 volts of electrical potential (electric eel, *Electrophorus voltaic* Linnaeus, 1766). Other fishes, such as sharks and knifefish, can sense weak electrical fields emitted by other animals to locate prey using unique electroreceptors [184]. A few examples also include aquatic mammals, e.g., Guiana dolphin, as well as terrestrial animals such as the honeybee, platypus, and echidna. The honeybee generates an electrical charge via a fast-rate wing beat; this charge is transferred to a flower so that other bees can detect the effectuated foraging [185,186]. Echidna and platypus possess numerous electroreceptors on their snouts detecting water currents and allowing them to locate prey without using their eyes.

For many decades, electroperception has intrigued both biologists and engineers for the understanding of its mechanism, as well as for the transfer of its working principle to technical devices. Electroperception in fish was discovered and described in detail by Lissmann and Machin [187]. Subsequently, different studies were carried out to realize artificial biomimetic and bioinspired electrosenses, particularly in robotics, mimicking the electrosensory system and swimming mechanics of electric fish [188,189]. Indeed, biomimetic electrosense can aid the robotic navigation, detection, and discrimination of objects, as well as environmental mapping, i.e., typical tasks for which fish use electrosensing [187,188,189]. However, based on an electric field generation, artificial abstraction and biological electrosense show important differences: for example, the distribution of the electric field isopotential and number of sensors (30 of artificial system *versus* 10,000 of knifefish) [189].

Another renowned way in which electricity is used by animals is the attachment ability of the gecko. The gecko lizards (infraorder Gekkota) can climb walls and other surfaces due to the unique micro- and nanoscopic keratin foot fibrillar structures (setae and spatulae) [190]. Due to this structural arrangement, the gecko foot molecules adhere to the surface through Van der Waals forces [20,190]. Each hair produces a force ≈ 10^−7^ N; however, millions of hairs create a more powerful adhesion of the order of ≈10 N cm^−2^ [191]. The creation of adhesives that mimic the gecko mechanism is tempting and, as the size of the adherent object increases, the attachment effect becomes more challenging. Geim et al. [191] reported a prototype of a ‘gecko tape’ made by the microfabrication of dense arrays of flexible plastic pillars. They discussed that the adhesive force varied linearly with the surface area contact, proving that their experiment could be scaled up; thus, larger areas of this gecko tape could support heavier objects. For example, human palms have a total area of more than 200 cm^2^ and, if covered by such a material, would be able to support the weight of an average human. In support of their thesis, an available amount of gecko tape was used to support the weight of a suitably sized toy. Nonetheless, a prototype able to support a human size was not provided and, indeed, is still a challenge. Different synthetic adhesive materials with micro- and nanofibrils have been designed [192,193] and, although a good performance is demonstrated at small scales, the relevant problems of scaling force emerge beyond 1-centimetre-square contact areas [194]. Numerous studies have been carried out to understand the scaling of biological adhesion. Some revealed how the enhancement of the adhesive force as the organism size increases is not achieved through a simple proportional expansion of the pad area. This is also confirmed by the evaluation that its positive allometry (i.e., proportional expansion of the pad area with respect to the entire body) among different taxa is drastically limited by the body plan and its phylogenetic constrains [195]. Hence, other variables need to be taken into consideration. In particular, the scaling of adhesive mechanisms in organisms is dependent on different aspects regarding the attachment structures and substrates. One of the main contributions in scaling adhesion is the ratio between the surface area (e.g., toepad for a gecko) and the compliance of the adhesive system in the loading direction [196]. As the organismal size increases, the adhesive system becomes stiffer (minimizing compliance), increasing the maximum adhesive force. Thus, with respect to smaller geckos, larger geckos have a stiffer adhesive system that increases their ability of hanging and climbing. In biomimetics, this can represent another challenge: synthetic materials must be soft to increase the contact to the substrate, but also stiff to achieve high loads. However, soft materials can create a more extensive contact, but have a high compliance when loaded, whereas stiff materials have less compliance but cannot create large scale contact [194]. This constraint is overcome in different organisms (e.g., from flies to geckos) by developing attachment surfaces with unique fibrillar structures. These increase the contact area through a “contact splitting” and provide a low compliance in the loading direction, aligning them under shear loads [194,197]. In this regard, Arzt et al. [198] reported how the attachment ability of different organisms (flies, beetles, and geckos) based on the interaction of setae (or other fibrillar and patterned surfaces structures) to the substrate varied according to their size. The hypothesis was that the areal density of these attachment structures increased with the increase in body mass. Thus, the adhesion ability is enhanced by dividing the surface area into finer sub-contact areas and, consequently, multiplying the number of single contacts (i.e., contact splitting) [199,200]. Accordingly, flies and beetles adhere with attaching elements of micrometric dimension, whereas geckos increase their density by downscaling these elements into numerous sub-micrometric setae to ensure a stronger adhesion. However, this study is disputed since it assumes that all taxa evolved independently. Conversely, taking into consideration that some species are more closely phylogenetically related than others, the identified correlation between the setae morphology and body size became less significant [200]. In all cases, the contact splitting strategy also has a limitation: as the pad area increases, the role of the setae discretization decreases, since it enhances contact only at the micron or sub-micron length scale [194,197]. Therefore, while the presence of fibrillar structures contributes to the attachment ability of these animals, the copy of its form does not allow adhesion to human scales. Barlett et al. [194] proposes that the key to designing scalable reversible adhesive materials is the development of materials able to create a true contact area while minimizing compliance in the loading direction. Accordingly, they realized adhesive materials using fabric fibres with non-patterned reversibly adhesive elastomer surfaces, in which the fabric fibres provide stiffness in the direction of loading and the elastomer layers provide extensive contact. The non-patterned characteristic highlighted that the specific contacting geometry was negligible. The experimental tests showed how the adhesive ability was effectively scaled up from areas of 1 to 100 cm^2^ with no observed physical or manufacturing constraints limiting the ultimate size of the adhesives. Hence, the sub-surface structures maintaining an integrated stiffness could play an important role in the future realization of human-scale adhesives. Finally, the attachment substrate could also play an additional role, and some forms of adhesion are shown to be dependent on the substrate roughness amplitude [197].

### 2.4. Acoustics

Sound is a vibration propagating as an acoustic wave through a gas, liquid or solid. Sound production, dispersion and reception in animals is investigated by bioacoustics, i.e., the disciplinary combination of biology and acoustics [201,202]. Animals use acoustic and vibrational senses mainly to monitor their environment and to communicate with conspecifics. Interestingly, observations demonstrated that small animals generally use high frequencies for communication, whereas large animals use low frequencies [201]. Indeed, physical scaling laws are also present in this field since frequency is related to the physical properties of sound-producing mechanisms. Considering animals differing only in size, the vibration frequency of their sound-producing organ depends on the linear dimensions of the vibrating structure, which are proportional to the linear variation of the animal size, and on the density and elastic modulus of the material from which it is made. The frequency is inversely proportional to the linear size of the animal and f ∝ M^−1/3^, where M is the animal mass [201]. Regarding the sound detection, the conversion mechanisms of vibrations to neural impulses are similar in different animal classes; however, there are some specializations: from narrow band hearing, to detect predators (e.g., caterpillars detecting wing beats of wasps) and communicating with conspecifics; to a broad frequency range, to detect both the lower frequencies of larger predators and the higher frequencies of smaller prey [201].

In terrestrial and aquatic organisms, sound production systems and passive acoustic transductors (ears) developed in multiple forms and sizes during evolution (for a detailed review see [201]), as well as, related strategies to optimize function. These strategies inspired interesting biomimetic applications in different fields, particularly in the reduction in transport noise.

Diverse species have adapted to sound reduction, particularly during the motion approach to their prey. Many owl species (order Strigiformes) can silently approach their prey due to specialized feathers generating a low frequency noise below 2 kHz, which is in the hearing range of its prey (2 kHz to 20 kHz) [203,204]. Noise created by a moving object through air is mostly generated at its edges; accordingly, owls have wing feathers with flexible fringe edges able to minimize this noise-generated turbulence [203,205].

In 1970, based on this strategy, the effect of leading-edge serration was scaled and tested for its ability to reduce aerofoil tonal noise from helicopter blades [206,207,208]. Subsequently, low noise aerofoils and wind turbines with serrated patterns at the leading edges were developed [208]. In this regard, DinoTail^®^ Next Generation realized a retrofit for wind turbines applied to the trailing edge of blades by adding fine combs inspired by the flexible owl wing fringe edge. The fine combs resulted in over 10% noise reduction at all wind speeds without a loss of power.

**Figure 4:**
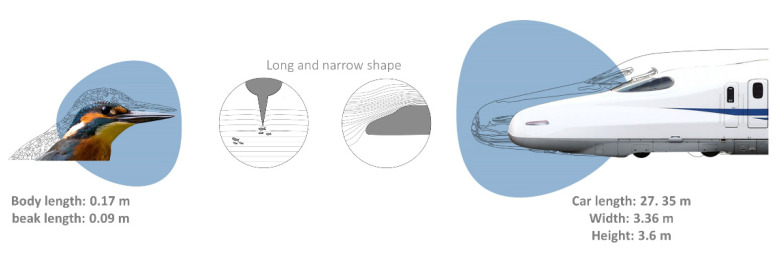
Biomimetic scaling of noise reduction. Kingfisher’s beak was transferred into the design of high-speed trains. These trains became quieter and more efficient requiring 15% less energy while traveling even faster than before. Train image adapted from [212].

Another excellent example is the beak of the kingfisher (family Alcedinidae), shaped as a long and narrow cone, able to enter the water without creating a compression wave below the surface and a noisy splash above. This is achieved by the reduction in the surface area or the modification resistance to water upon entry, as well as, by gradually enlarging the cross-section of the beak penetration, keeping the fluid flowing smoothly around it [209,210]; allowing kingfishers to silently reach fish. This strategy was used by the Japanese engineer Eiji Nakatsu to redesign high speed trains, avoiding the noise generated by moving from the open air to closed tunnels, caused by the change in air resistance. By mimicking the kingfisher beak, the nose of the train was more silent and efficient, using 15% less energy while traveling faster than before [211] (Figure 4).

Numerous organisms can amplify and manipulate acoustic waves to perceive, localize, and intra- and interspecifically communicate. Famous examples are the bat and dolphin biosonar systems, which allow them to navigate and forage at night or in murky deep waters [202,213,214].

In aquatic environments, cetaceans (infraorder Cetacea) use a variety of calls including clicks, whistles and songs for communication, social interaction, navigation, and foraging [215,216]. Each call is produced within a broad frequency that continuously changes, transmitting information and compensating interference that can occur underwater, such as echoes and noise. These strategies are used to develop different underwater acoustic communication systems [216]. Indeed, transmitting data underwater is challenging due to motion, noise, limited bandwidth, and variable delays reducing the transmission quality and speeds needed for underwater sensor networks, communication, and defence applications (see [216] for a review). The S2C technology was developed by EvoLogics to continuously spread underwater signals using a wide range of non-interfering frequencies, enabling a successful decoding of signals in harsh and noisy environments [217]. This system allows the detection of underwater earthquakes and fast tsunami early warning systems [217].

## 3. Discussion

The present work aimed to review a part of the existing organismal knowledge and biomimetic technology concerning scale-relating principles. Although not exhaustive due to the vastness of the fields considered, the examples reported are emblematic in biology and biomimetic design and intend to highlight the main scale problems.

By exploring different field of physics, it is possible to notice how organismal design is drastically affected by scale dimension. Additionally, diverse technical examples demonstrated where scaling can be effectively performed and, contrarily, where it is limited. Effective scaling can be seen in artifacts inspired by mechanic and dynamic biological principles, e.g., building constructions, robotics, aerospace, and transportation. In optics, pigmentary colours and visual effects have historically found interesting applications at a large scale, e.g., the U.S. Navy ships and aircrafts. In contrast, the scalability of structural colours is limited since the visual effect strictly depends on the dimensional features of the structure, which must be comparable with the wavelength of the electromagnetic radiation with which it interacts. Similar constrains in scalability can be found in electric phenomena, being part of electromagnetism, and in acoustic phenomena, being a wave form.

Based on current literature, several key parameters can be identified in scaling transfer. They consist of the main variables needed to be taken into consideration when comparing the scale of the biological model with the final application.

In mechanics, static and kinematic mechanical notions must be taken into consideration for the identification of the key parameters. As seen for a giant sequoia, with respect to a smaller tree (see Section 2), the main variable to consider in the scaling up process is the self-weight, and thus the impact of gravity [68]. Accordingly, the general key parameters to consider in scaling constructions are: mass, geometry, material properties, and applied forces (gravitational or other forces, e.g., seismic). In dynamics, additional parameters should be taken into consideration. Kinematic analyses are often used to quantify the performance of animal dynamic functions [3,5,50]. These consist of discerning patterns in the displacement of one or more components, calculating key variables such as velocities, accelerations, and movement timing. The correlation between body size and kinematic performance was investigated in different organisms and these studies can be considered as guidelines to understand the specific dynamic function variation in relation to scaling [218,219]. As previously mentioned, Reynolds numbers are an important quantity to take into consideration in the scaling of swimming and flying systems, since they involve a tough interaction with the fluid (i.e., water or air).

In optics, wavelength and amplitude are the main parameters to be considered. Wavelength determines the position of an electromagnetic wave within the electromagnetic spectrum. Wavelength is inversely related to frequency; thus, increasing frequency (or decreasing wavelength). Electromagnetic waves can be classified in: radio waves, microwaves, millimetre waves, infrared radiation, visible light, ultraviolet radiation, X-rays and gamma rays [139,220]. Each wave interacts differently with matter [139]. Moreover, in the visual systems, different wavelengths are generally associated with different colours, and their amplitude determines their brightness. Other key variables to be considered are the characteristics of the surface the wave interacts with (i.e., dimension, geometry, and material). For example, micro and nanostructures can reflect, absorb, and manipulate visible light as seen in structural colours, whereas in light perception, changes in the lens-shaped surface and photoreceptor characteristics (e.g., types, density, and disposition) determine different visual acuity and sensibility [221].

In electricity, relevant parameters include charge amount, current intensity, voltage, and field amplitude. Moreover, emitted field structure (e.g., signal shape, frequency, and amplitude), sensing layout (number and position), distance between source and object, dielectric properties and geometry of object, and dielectric properties of surrounding fluid (e.g., water) play a crucial role. In this regard, useful guidelines have been provided in the literature, especially regarding the development of bioinspired electrosense devices [189,222].

In acoustics, as seen for light waves, the main parameters to take into consideration are the physical properties of sound waves to which various aspects of sound perception are associated [201,202]. In particular, the frequency (Hz) of a sound wave is associated with the sound pitch and range of perception. In humans, the audible range of sound frequencies is between 30 and 20,000 Hz [221]. Conversely, wave amplitudes determine the loudness (decibels dB) of a given sound: louder sounds are determined by higher amplitudes. Other parameters to be considered are related to physical characteristics (e.g., dimension, geometry, and materials) of the interactive surfaces or devices of perception with which the sound waves interact [201,202,221]. In this regard, different types of passive bioacoustics transductors are present in the animal kingdom. Ears act as reception antennas that can intercept and focus sound waves from the environment. Different dimensions and geometries adapt to intercept different types of sound. For examples, the narrow and elongated ears of common hare adapt to effectively perceive sounds generated on the ground [221]. Other unique dimensions, forms, dispositions, and mobilities of ears (e.g., owls and bats), combined with a related signal elaboration, also provide organisms with different strategies to hear or locate prey and predators [201,221]. In the case of echolocation, like electrosense, other characteristics to be considered are the distance between source and object, intensity and form of the emitted echolocation beam, and sound propagation in fluid (e.g., air or water). Particularly, the sound intensity from a source of sound to an object obeys the inverse square law [221]. Thus, the intensity decreases with the square of the radius of action (this is also true for human radar and sonar). Moreover, the form of the sound beam is usually optimized to be directional to the target object (e.g., preys). In this regard, different specializations are present in organismal design, such as the nose shape of chiropters and the melon structure of the odontocete adapted to effectively direct their echometric emission [221,223].

Lastly, as a general consideration, materials and their specific mechanic, optic, electrical, and acoustic properties should be considered in all types of scaling transfer.

Significant issues regarding biomimetic transfer are related to the difference between organisms and artefacts: the large sizes and fast speeds of the engineered systems drastically differ from biological systems, which are usually small and relatively slow. Organisms range from 400 nanometres to 90 metres, and technical devices from nanometres to kilometres. In building constructions, size, materials, and external loads are extremely different from the mechanical support systems of organisms. Biological structures are usually resistant to large forces with deflections of soft and flexible parts, which are not acceptable in building construction (e.g., Television tower and grass blade) [2]. Moreover, as discussed by Fish et al. [107]: “jet aircraft carry greater payloads and fly faster at higher altitudes than do small birds; race cars move faster over land than cheetahs, gazelles, or racehorses”. In the technological transfer, these discrepancies could determine scale limits. Thus, a specific contextualization and optimization of the biomimetic solutions is required and can be performed through specific methods and tools, such as a “computer-aided optimization” (CAO), knowledge database and algorithms [60,224].

Hence, the problem of scale in biomimetics could be reduced by finding areas of overlap in the size and performance between a biological structure and its technical applications [10] or by abstracting biological principles to then be translated, redesigned, contextualized, optimized, and successfully applied as effective solutions [12]. The arising questions are: when does this abstraction become too distant from the biological working principle to not be considered as a proper biomimetic translation? Can the transformation of the biological process to other physical-chemical processes, in the achievement of the same biological functions circumventing the scale problem (as encouraged by Perez et al. [19]), still be identified as a biomimetic process? Does the main emphasis lie in the reproduction of the final biological function or in the process itself?

In conclusion, despite the significant scientific progresses in the development of innovative bioinspired technologies from the macro- to nanoscale, the scaling problem is a crucial and overlooked aspect that requires more attention in the practice of biomimetics. The transfer of functional strategies can be carried out if the rules of similarity are respected in other scales. Future research should be carried out to establish some guidelines in the scaling transfer for each field of knowledge, providing effective analytic models and definitions of the similarity limit in each biomimetic transfer. For example: wanting to realize a shell structure inspired by the echinoid skeleton tessellation, how should the ratio of its size be reproportioned based on the considered applied loads? When is the biological strategy transfer compromised by the change in scale? To truly understand the applicability of a specific biomimetic strategy, these aspects should be evaluated and compared from the biological scale up to the technical one.

This review launches an initial discussion of this complex topic, which remarkably affects the biomimetic process from a conceptual to an applicative perspective. In biology, the scale problem of the organismal design is profoundly debated, and numerous studies continuously reveal novel findings and knowledge. In biomimetics, the highlighting of this aspect is useful and sometimes crucial for the effectiveness of the transfer.

Too often, the scalability is implemented naively and automatically, leading to a misrepresentation of the evolutive meaning of the biological characteristics and in consequence to an erroneous and/or distorted development of biomimetic design projects. Once again, nature can teach us the correct way to create innovation, always considering the opportunities and constraints of our physical world.
